# Clonal evolution patterns in acute myeloid leukemia with *NPM1* mutation

**DOI:** 10.1038/s41467-019-09745-2

**Published:** 2019-05-02

**Authors:** Sibylle Cocciardi, Anna Dolnik, Silke Kapp-Schwoerer, Frank G. Rücker, Susanne Lux, Tamara J. Blätte, Sabrina Skambraks, Jan Krönke, Florian H. Heidel, Tina M. Schnöder, Andrea Corbacioglu, Verena I. Gaidzik, Peter Paschka, Veronica Teleanu, Gudrun Göhring, Felicitas Thol, Michael Heuser, Arnold Ganser, Daniela Weber, Eric Sträng, Hans A. Kestler, Hartmut Döhner, Lars Bullinger, Konstanze Döhner

**Affiliations:** 1 Department of Internal Medicine III, University Hospital of Ulm, Ulm, 89081 Germany; 2 Department of Internal Medicine II, Hematology and Oncology, Friedrich-Schiller-University Medical Center, Jena, 07743 Germany; 3 Leibniz-Institute on Aging, Fritz-Lipmann-Institute, Jena, 07745 Germany; 4 Institute of Cell & Molecular Pathology, Hannover Medical School, Hannover, 30625 Germany; 5 Department of Haematology, Haemostasis, Oncology, and Stem Cell Transplantation, Hannover Medical School, Hannover, 30625 Germany; 6 Institute of Medical Systems Biology, Ulm University, Ulm, 30625 Germany; 7 Department of Hematology, Oncology and Tumorimmunology, Charité University Medicine, Berlin, 13353 Germany

**Keywords:** Acute myeloid leukaemia, Cancer genetics, Acute myeloid leukaemia

## Abstract

Mutations in the *nucleophosmin 1* (*NPM1*) gene are considered founder mutations in the pathogenesis of acute myeloid leukemia (AML). To characterize the genetic composition of *NPM1* mutated (*NPM1*^mut^) AML, we assess mutation status of five recurrently mutated oncogenes in 129 paired *NPM1*^mut^ samples obtained at diagnosis and relapse. We find a substantial shift in the genetic pattern from diagnosis to relapse including *NPM1*^mut^ loss (*n* = 11). To better understand these *NPM1*^mut^ loss cases, we perform whole exome sequencing (WES) and RNA-Seq. At the time of relapse, *NPM1*^mut^ loss patients (pts) feature distinct mutational patterns that share almost no somatic mutation with the corresponding diagnosis sample and impact different signaling pathways. In contrast, profiles of pts with persistent *NPM1*^mut^ are reflected by a high overlap of mutations between diagnosis and relapse. Our findings confirm that relapse often originates from persistent leukemic clones, though *NPM1*^mut^ loss cases suggest a second “de novo” or treatment-associated AML (tAML) as alternative cause of relapse.

## Abstract (web summary)

*NPM1* gene mutation is a founding event in acute myeloid leukaemia. Here, the authors find that at relapse, some patients lose the *NPM1* mutation and show distinct mutational and gene expression patterns, highlighting a potential route for relapse.

## Introduction

One of the most common mutations (mut) in AML involves the *NPM1* gene, which is present in about one third of AML pts^1^. *NPM1*^mut^ AML is characterized by distinct biological and clinical features and pts with the *NPM1*^mut^ and no *FLT3* internal tandem duplication (ITD) or low *FLT3*-ITD levels have a good response to induction chemotherapy and a favorable prognosis. Based on these findings *NPM1*^mut^ AML was included as an entity in the World Health Organization classification 2016 and the *NPM1*^mut^/*FLT3*-ITD genotypes stratified by the ITD allelic ratio were integrated in the risk stratification of the European LeukemiaNet (ELN) recommendations, subdividing AML in subsets with highly distinct prognosis^2–4^.

For a long time *NPM1*^mut^ was considered a founder event, because it is usually maintained at relapse. The consideration of *NPM1*^mut^ as a founder event in AML is further supported by the distinct morphological and clinical presentation associated with this subtype of AML. However, recent studies have shown that *NPM1*^mut^ occurs rather late, due to its absence in preleukemic hematopoietic stem cells (HSCs)^5,6^. Moreover, in ~10% of relapsed pts *NPM1*^mut^ is lost while further chromosomal and molecular changes are acquired^7–9^.

The recently identified preleukemic mutations in *DNMT3A*, *TET2*, *ASXL1*, *IDH1*, and *IDH2* often persist at remission due to persistent clonal hematopoiesis^5,10^. Preleukemic mutations were also found to be present in non-leukemic T cells of AML patients, at the time of diagnosis and they have been identified in individuals without hematologic malignancy or who were unselected for cancer or hematologic phenotypes in an age related-manner^11^. Age-related clonal hematopoiesis is a common condition that is associated with an increased risk to develop hematologic cancer^12–15^. Indeed, co-occurring mutations in DNA methylation or hydroxymethylation genes (*DNMT3A*, *IDH1*, *IDH2*^R140^, and *TET2*) are frequent amongst *NPM1*^mut^ leukemias and found in ~73% of pts^6^.

In our previously investigated cohort of 53 *NPM1*^mut^ AML pts, we also described this frequent co-occurrence of preleukemic mutations^7^. Here, the majority of relapsed leukemias showed clonal evolution with a clear relationship of relapse and diagnostic leukemia clones by acquisition of additional genetic lesions, or more commonly the relapse clone arose from a common ancestral clone, which was also shown by other studies^7,16,17^. Interestingly, in our study we identified five pts with *NPM1*^mut^ loss at relapse showing a shift in genetic lesions between the diagnosis and relapse sample; clinically, these patients had a significantly longer time to relapse compared to *NPM1*^mut^ persistent pts (33.7 months versus 8.6 months, *p* = 0.03), and none responded to salvage therapy. To date, it is still unclear whether in these cases clonal evolution resulted in loss of *NPM1*^mut^ or whether the initial leukemia clone was cured and a new leukemia developed on the basis of dominant clonal hematopoiesis with persisting preleukemic mutations. This new leukemia could then also be considered as therapy-related AML (tAML) that evolved from a common pre-leukemic clone.

To gain further insight into the genomic evolution of *NPM1*^mut^ AML, we have extended our previous cohort and assess mutational status of five genes by conventional mutation analysis in paired samples obtained at diagnosis and relapse from 129 *NPM1*^mut^ pts. For a subgroup of pts a more detailed analysis comprising the mutational status of nine genes and comprehensive SNP microarray profiling is performed. Finally, for in-depth analysis we sequence the exome of ten pts with persistent *NPM1*^mut^ and ten pts with loss of *NPM1*^mut^ and perform RNA-Seq analysis for selected *NPM1*^mut^ persistent and loss cases. Findings are confirmed by targeted deep-sequencing and flow cytometry based single cell protein expression analysis.

## Results

### Genomic characterization of clonal evolution in *NPM1*^mut^ AML

**Fig. 1:**
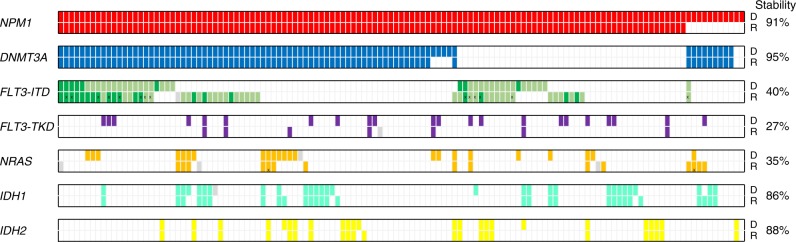
Incidence of mutations in 129 paired (diagnosis/relapse) *NPM1*^mut^ pts. Colored bars indicate the presence of a mutation, white bars represent wild-type, data not available is indicated by a gray bar. Light and dark green bars illustrate heterozygous and homozygous *FLT3*-ITD mutations, respectively. Bars marked by X illustrate different mutation types found in the diagnosis and relapse sample. Stability was calculated by the number of mutations that persisted at relapse divided by all mutations present at diagnosis. D, diagnosis; R, relapse

Paired samples at diagnosis and relapse from 129 *NPM1*^mut^ AML pts were assessed for clonal evolution-associated mutations in the most recurrently mutated genes (*FLT3*, *DNMT3A, IDH1, IDH2, NRAS*) by conventional mutation analysis as previously reported^7^. At diagnosis, 83 pts (64%) harbored concurrent *DNMT3A*^mut^, 40 pts (31%) *FLT3*-ITD, 22 pts (17%) *FLT3*-tyrosine kinase domain (TKD)^mut^, 23 pts (18%) *NRAS*^mut^, 29 pts (23%) and 24 pts (19%) *IDH1*^mut^ and *IDH2*^mut^, respectively (Fig. 1). In addition, we screened a subgroup of pts for *MLL*-partial tandem duplications (PTD) and mutations in *ASXL1*, *TP53* and *RUNX1*. None of the pts analyzed had a *MLL*-PTD, *ASXL1*^mut^, *TP53*^mut^ or *RUNX1*^mut^ at the time of diagnosis (Supplementary Fig. 1 and Supplementary Table 1).

At relapse, a shift in the mutation pattern was found in 76 pts (59%, Supplementary Table 2). While *NPM1*^mut^ was lost in 11 pts (9%), *DNMT3A*^mut^ persisted in 79 of 83 pts (95%) and thus was the most stable mutation at relapse. Sixteen of 40 pts (40%) had the identical *FLT3*-ITD, whereas 14 pts (35%) showed a distinct *FLT3*-ITD clone indicated by a change of the ITD length, and 10 pts (25%) lost the *FLT3*-ITD at relapse. Gain of a *FLT3*-ITD clone was detected in 23 of 128 cases (18%). Similarly, *FLT3*-TKD^mut^ and *NRAS*^mut^ were rather unstable and lost in 16 of 22 pts (73%) and 15 of 23 pts (65%), respectively. On the contrary, *IDH1*^mut^ and *IDH2*^mut^ were relatively stable, with persistence of the diagnosis mutation in 25 of 29 pts (86%) for *IDH1*^mut^ and 21 of 24 pts (88%) for *IDH2*^mut^. For *MLL*-PTD, *ASXL1*^mut^, *TP53*^mut^ and *RUNX1*^mut^, none of the pts with *NPM1*^mut^ loss had a mutation at diagnosis; however, at relapse four pts gained an *MLL*-PTD, one an *ASXL1*^mut^, and three a *RUNX1*^mut^ (Fig. 1 and Supplementary Fig. 1).

**Fig. 2:**
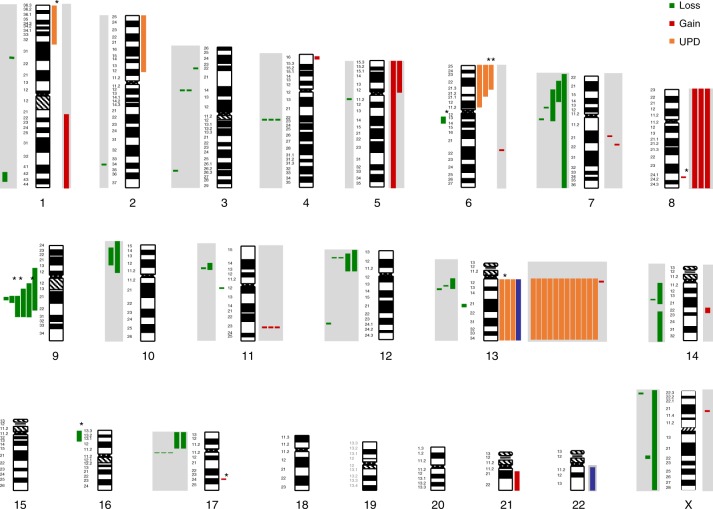
CNAs and UPDs identified at diagnosis and relapse by SNP profiling. Bars next to each chromosome indicate genomic gain, loss, and uniparental disomy (UPD). The length of the bar indicates the respective genomic region affected by the aberrations. Bars highlighted by a grey box indicate relapse specific aberrations. Bars marked by a star indicate genomic changes which are lost at relapse

Following up on our previous report^7^, we extended single nucleotide polymorphism (SNP) microarray profiling to a total of 77 *NPM1*^mut^ pts [cytogenetically normal (CN) karyotype, *n* = 73; del(9q), *n* = 4]. In addition to these findings also seen by cytogenetics, the analysis of diagnostic samples confirmed copy number alterations (CNAs) and uniparental disomy (UPD) previously associated with CN *NPM1*^mut^ including del(4)(q22.1), del(12)(p13.2), del(17)(q11.2), gain of chromosome 8 (trisomy 8) and UPD affecting chromosomal arm 13q (Fig. 2 and Supplementary Table 3). In the extended cohort, novel findings at diagnosis included del(11)(q12.3), gain(8)(q24.21), and a trisomy 21 as well as UPDs affecting the chromosomal arm 6p in 3 pts (Fig. 2). At relapse, the previously reported deletions of 12p13 and 17q11.2, gain(11)(q23.3), trisomy 8 and UDP 13 could also be confirmed. In addition, SNP-array analysis identified nine non-recurrent CNAs [del(5)(q11.2q11.2), del(10)(p11.21p15.3), del(10)(p12.31p14), del(14)(q11.2q21.3), del(14)(q23.1q32.33), del(X)(p22.32q26.1), gain(1)(q21.1q44), gain(5)(p11p15.33), gain(14)(q22.1q22.2)], as well as one UPD affecting the long arm of chromosome 21 (Fig. 2). The incidence of chromosomal aberrations (deletion, gain, or UPD) was 3.4 times higher at relapse (diagnosis *n* = 23, relapse *n* = 79) as shown previously^7^.

Overall, we detected a gain of CNAs and/or UPDs at relapse in 66.7% (4/6) of *NPM1*^mut^ loss and 43.7% (31/71) of *NPM1*^mut^ persistent samples. Patients with gain of CNA and/or UPD also acquired more mutations at relapse based on mutations detected with WES (median 7.5 vs 2; pts with gain of CNA and/or UPD, *n* = 8; pts without gain of CNA and/or UPD, *n* = 5). This supports our observation that *NPM1*^mut^ loss pts are less stable in terms of their mutational profile compared to *NPM1*^mut^ persistent pts. Interestingly, 10/11 *NPM1*^mut^ loss pts were in the favorable ELN 2017 risk group at the time of diagnosis while only one was in the intermediate risk group.

### Comparative analysis of *NPM1*^mut^ loss and persistent pts

To gain further insight into the relapse mechanism of pts with loss of the *NPM1*^mut^, we performed WES of paired samples (diagnosis, remission and relapse) from 10 *NPM1*^mut^ loss and 10 *NPM1*^mut^ persistent pts. The mean depth of the covered exome was 91 reads for diagnosis, 92 reads for remission, and 89 reads for relapse samples (Supplementary Table 4). At diagnosis, we identified on average 9.7 mutations per case (8.8 in *NPM1*^mut^ loss and 10.6 in *NPM1*^mut^ persistent pts) and at relapse 11.4 mutations per case (9.4 in *NPM1*^mut^ loss and 13.4 in *NPM1*^mut^ persistent pts).

**Fig. 3:**
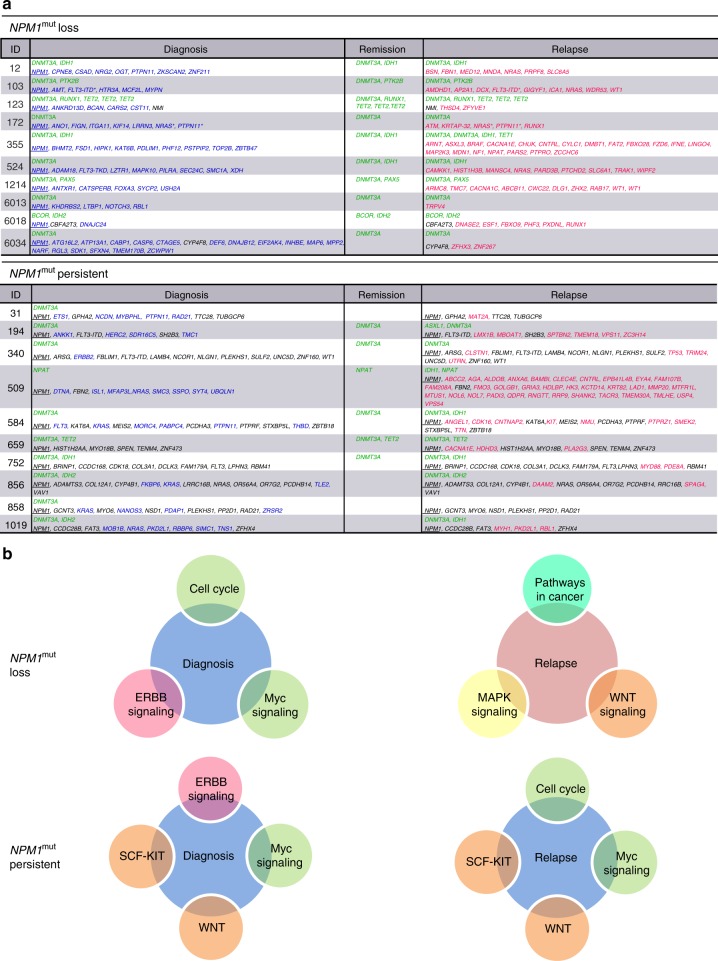
Mutational map and enriched pathways in diagnosis/relapse pairs of *NPM1*^mut^ loss and persistent pts. **a** Mutational profile of 10 *NPM1*^mut^ loss and 10 *NPM1*^mut^ persistent pts found by WES. Colors indicate the varying occurrence of mutations during disease progression, including mutations which persist during remission (preleukemic/germline mutations; [green]), mutations which are solely present in the diagnosis sample (blue), relapse specific mutations (red) and mutations which are shared between the diagnosis and relapse sample (black). **b** Enriched pathways in diagnosis and relapse samples from *NPM1*^mut^ loss and *NPM1*^mut^ persistent pts based on mutated genes

In all *NPM1*^mut^ loss pts, WES identified at least one previously described preleukemic mutation with the majority carrying *DNMT3A*^mut^ (9/10 pts, Fig. 3a and Supplementary Data 1)^5,11,12^. Notably, all preleukemic mutations persisted with a similar variant allele frequency (VAF) in remission and relapse (Supplementary Fig. 2a). In addition, we identified mutations persisting at remission in genes not yet associated with clonal hematopoiesis such as *PTK2B* and *PAX5*. Apart from these persisting mutations, diagnosis and relapse samples had distinct mutational patterns and shared almost no somatic mutation. Thus, in *NPM1*^mut^ loss pts not only *NPM1*^mut^ but also all other somatic mutations were lost, thereby suggesting clearance of the initial leukemic clone and occurrence of a novel leukemia at the time of relapse.

On the contrary, in all *NPM1*^mut^ persistent pts at least three common mutations (mean 7.6) between diagnosis and relapse were found (Fig. 3a and Supplementary Data 2). Furthermore, preleukemic mutations persisted in only six of the 10 pts during remission, and in these cases the VAF was lower compared to *NPM1*^mut^ loss pts (Supplementary Fig. 2b). These findings suggest that the preleukemic clone is not yet fully dominating hematopoiesis in all *NPM1*^mut^ persistent pts. However, reappearance of preleukemic mutations in conjunction with the persistence of *NPM1*^mut^ and other mutations at the time of relapse suggests that a leukemic stem cell clone survived initial chemotherapy and gave rise to relapsed disease shaped by clonal evolution.

We performed deep amplicon sequencing to survey diagnosis samples from three *NPM1*^mut^ loss and three *NPM1*^mut^ persistent pts for relapse specific mutations at the time of diagnosis. First, we wanted to see whether relapse specific mutations in *NPM1*^mut^ persistent cases were already present at diagnosis, and second we wanted to rule out the possibility that a minor *NPM1*^mut^ loss subclone was already present at this time point. Coverage for the respective variant positions ranged from 423 to 16031 reads (mean 4224, median 3023). In none of the *NPM1*^mut^ loss diagnosis samples we were able to detect a relapse specific mutation (Table 1) while in *NPM1*^mut^ persistent pts we detected a relapse specific mutation in 2 out of 3 pts with VAFs of 0.6 and 0.17%, respectively (*TP53*^mut^ in ID340 and *IDH1*^mut^ in ID1019).

**Table 1:** Relapse specific mutations detected in diagnosis samples with UltraDeep-sequencing

Patient ID	Chr	Start	End	Ref	Alt	Gene	AAChange	VAF % relapse (WES)	VAF % remission (WES)	VAF % diagnosis (WES)	Found at diagnosis (UltraDeep-seq)	Coverage (UltraDeep-seq)	VAF % (UltraDeep-seq)
103 L	chr11	32417907	32417907	–	CCGA	WT1	A382fs	13.54	0	0	NO	9660	0
103 L	chr1	115258744	115258744	C	T	NRAS	G13D	31.89	0.45	0	NO	8062	0
103 L	chr2	25463286	25463286	C	T	DNMT3A	R736H	25	37	51.35	YES	1128	29.8
172 L	chr12	112888211	112888211	A	C	PTPN11	E76A	14.29	0	0	NO	5244	0
172 L	chr21	36171600	36171600	–	A	RUNX1	S322fs	50	0	0	NO	2207	0
172 L	chr2	25457243	25457243	G	A	DNMT3A	R882C	38.46	36	38.46	YES	4531	48.5
172 L	chr1	115258744	115258744	C	T	NRAS	G13D	8	0	0	NO	9311	0
355 L	chr7	140476755	140476755	T	C	BRAF	I551V	26.47	0	0	NO	2282	0
355 L	chr2	25458596	25458596	T	A	DNMT3A	L859F	35.29	0	0	NO	1163	0
355 L	chr17	29662024	29662024	–	A	NF1	A1994fs	36.54	0	0	NO	3309	0
355 L	chr2	209113113	209113113	G	T	IDH1	R132S	40	33	43	YES	2736	45.1
355 L	chr2	25468187	25468187	A	C	DNMT3A	C497G	29.27	25	48.08	YES	646	44.8
340 P	chr2	25457242	25457242	C	T	DNMT3A	R882H	11	7	50	YES	1172	57.3
340 P	chr5	170837543	170837543	–	TCTG	NPM1	L287fs	40	0	nd	YES	698	36.5
340 P	chr13	28608244	28608244	–	1*	FLT3	2*	nd	0	nd	YES	10884	1.9
340 P	chr17	7577548	7577548	C	T	TP53	G206S	61.9	0	0	YES	5151	0.6
340 P	chr11	32417914	32417914	–	T	WT1	R380fs	50.53	0	32.14	YES	6096	38.9
584 P	chr5	170837543	170837543	–	TCTG	NPM1	L287fs	nd	0	nd	YES	553	40.3
584 P	chr2	25457252	25457252	T	C	DNMT3A	N879D	20	37	47.62	YES	1133	62.5
584 P	chr2	209113112	209113112	C	T	IDH1	R132H	31.94	0	0	NO	3550	0
584 P	chr4	55599321	55599321	A	T	KIT	D816V	30.9	0	0	NO	16031	0
1019 P	chr5	170837545	170837545			NPM1	L287fs	nd	0	nd	YES	423	21.7
1019 P	chr2	25457242	25457242	C	T	DNMT3A	R882H	68.75	0	66.67	YES	862	49.4
1019 P	chr2	209113112	209113112	C	T	IDH1	R132H	45.45	0	0	YES	4553	0.2

1* TCCCATTTGAGATCATATTCATATTCTCTGAAATCAACGTAG

2* E604delinsDYVDFREYEYDLKWE

*L* *NPM1*^mut^ loss patient, *P* *NPM1*^mut^ persistent patient, *VAF* variant allele frequency, *WES* whole exome sequencing, *nd* not detected with exome sequencing

To explore whether mutated genes perturb similar biological pathways in diagnosis and relapse samples, we performed gene set over representation analysis in *NPM1*^mut^ loss and *NPM1*^mut^ persistent pts at both time points. We found in both *NPM1*^mut^ loss as well as *NPM1*^mut^ persistent cases diagnostic mutations to be enriched for members of the ERBB and MYC signaling pathways (Fig. 3b and Supplementary Data 3). For *NPM1*^mut^ persistent cases diagnosis and relapse associated mutations showed a highly concordant mutational pattern with mutations being enriched for members of the MYC, SCF-KIT (stem cell factor) and WNT signaling pathways. In contrast, the mutational spectrum of *NPM1*^mut^ loss relapse samples affected very different pathways, such as MAPK signaling and pathways known to be play a role in cancer (Pathways in Cancer), although a more stem cell-like phenotype (WNT signaling) was seen in these cases (Fig. 3b).

**Fig. 4:**
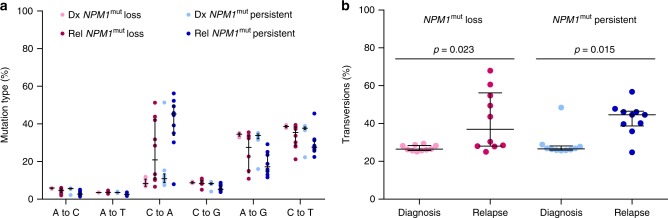
Mutation types before and after chemotherapy in *NPM1*^mut^ loss and persistent pts. **a** Frequency of transversions (A to C, A to T, C to A, and C to G) and transitions (A to G and C to T). **b** Proportion of transversions in *NPM1*^mut^ loss diagnosis (rose) and relapse (pink) samples and *NPM1*^mut^ persistent diagnosis (light blue) and relapse (dark blue) samples

As previous chemotherapy might affect cancer mutation signatures, i.e. the type of single nucleotide variants (SNVs), we assessed the transversion frequency of mutations found in diagnosis and relapse samples from *NPM1*^mut^ loss and *NPM1*^mut^ persistent pts to evaluate whether *NPM1*^mut^ loss at relapse might be considered as tAML following cytotoxic therapy of the initial de novo AML. While Wong et al.^18^ recently investigated 22 pts with tAML and found no difference in transversion frequency compared to de novo AML and secondary AML (sAML), Ding et al.^17^ reported a strong increase of transversions after chemotherapy in relapse compared to primary tumors. Here, we saw an increase of mainly C to A transversions from diagnosis to relapse in both groups (Fig. 4a), (*NPM1*^mut^ loss: *p* = 0.023; *NPM1*^mut^ persistent: *p* = 0.014, two-tailed Mann–Whitney test, Fig. 4b).

### RNA-Seq of *NPM1*^mut^ loss and persistent pts

**Fig. 5:**
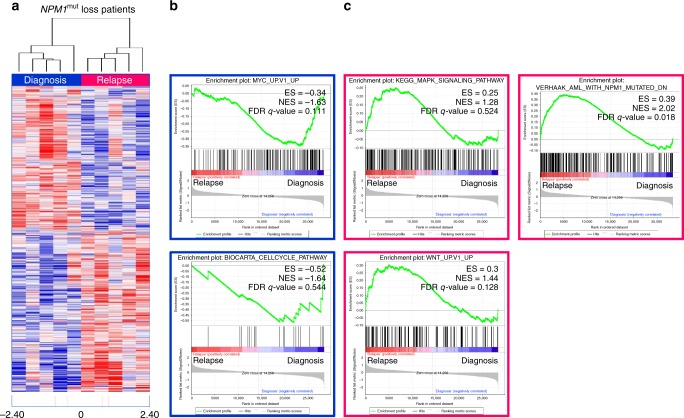
Heatmap of differentially expressed genes and GSEA of diagnosis versus relapse *NPM1*^mut^ loss samples. **a** Differentially expressed genes (significant at 0.05 level of the univariate test) from RNA-Seq were assessed using the ClassComparison option from BRB-ArrayTools. Blue indicates upregulation and red downregulation between groups. **b** Diagnosis samples show enrichment of MYC_UP.V1_UP and BIOCARTA_CELLCYCLE_PATHWAY gene sets. **c** Relapse samples were enriched in KEGG_MAPK_SIGNALING_PATHWAY, WNT_UP.V1_UP gene sets and genes which are downregulated in *NPM1*^mut^ samples (VERHAAK_AML_WITH_NPM1_MUTATED_DN). ES, enrichment score; FDR, false discovery rate; NES, normalized enrichment score

To determine whether specific mutational patterns in diagnosis and relapse samples of *NPM1*^mut^ loss pts have an impact on gene expression, we performed RNA-Seq. The expression of 28,922 genes above detectable expression levels (>1 read in at least one sample) was compared in a pairwise approach between diagnosis (*n* = 5) and relapse (*n* = 5) *NPM1*^mut^ loss samples. This resulted in 2141 differentially expressed genes (*p* ≤ 0.05) (Fig. 5a and Supplementary Data 4). Among these, we identified *HOXA10*, *HOXB6,* and *MEIS1*, an *NPM1*^mut^ associated homeobox (HOX) expression signature^19^, highly expressed in diagnosis samples, which was not seen in *NPM1*^mut^ loss relapse samples.

Next, we used the *NPM1*^mut^ signature from Verhaak and colleagues^19^, comprising a total of 18 genes, which can discriminate between *NPM1*^mut^ and *NPM1* wildtype (*NPM1*^wt^) AML, for hierarchical clustering of paired diagnosis and relapse samples from 5 *NPM1*^mut^ loss and 5 *NPM1*^mut^ persistent pts. This *NPM1*^mut^ signature grouped the relapse samples of the five cases that had lost the *NPM1*^mut^ at relapse (*NPM1*^wt^ cases) next to the 15 *NPM1*^mut^ samples. This *NPM1*^wt^ group is characterized by high expression of *APP*, *NRGN* and low expression of *HOXB5*, *HOXB6*, *HOXA9* and *HOXA10* (Supplementary Fig. 3).

Gene set enrichment analysis (GSEA) further supported our genomic WES based findings, as the gene expression signature of *NPM1*^mut^ loss relapse samples showed an enrichment of genes belonging to Pathways in cancer and more specifically of MAPK and WNT signaling, while in diagnosis samples pathways related to cell cycle and MYC signaling were affected (Fig. 5b, c and Supplementary Data 5). In addition, in relapse samples we found a significant up-regulation of genes, which have been shown to be down-regulated in *NPM1*^mut^ AML (FDR *q*-value = 0.018) (Fig. 5c)^19^. This demonstrates that relapse samples are *NPM1*^wt^ also on the functional level.

### CTNNB1 levels in *NPM1*^mut^ diagnosis samples

**Fig. 6:**
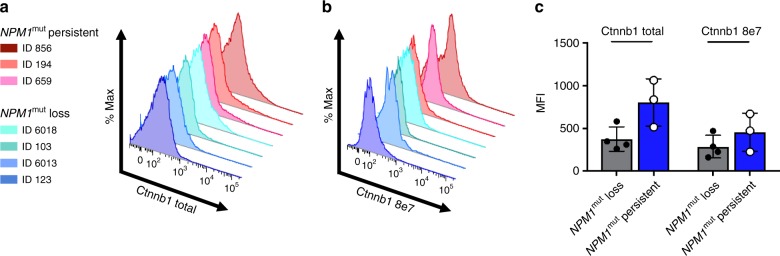
Flow cytometry of total and active CTNNB1 in diagnosis samples of *NPM1*^mut^ loss and *NPM1*^mut^ persistent pts. Higher expression of **a** CTNNB1 total, **b** CTNNB1 8e7 in 3 *NPM1*^mut^ persistent diagnosis samples compared to 4 *NPM1*^mut^ loss diagnosis samples, and **c** mean fluorescent intensity (MFI) including all data points, data is presented as mean ± s.d

To confirm stronger WNT signaling in *NPM1*^mut^ persistent diagnosis samples compared to *NPM1*^mut^ loss diagnosis samples, which was predicted by pathway analysis of mutated genes and transcriptomic analysis, we performed flow cytometry in three diagnosis samples of *NPM1*^mut^ loss and *NPM1*^mut^ persistent cases, respectively. Using an intracellular staining protocol for CTNNB1 (ß-Catenin), which is a key player in the WNT signaling pathway and crucial for leukemic stem cells^20^, we could confirm higher levels of both total CTNNB1 and active CTNNB1 in *NPM1*^mut^ persistent diagnosis samples compared to *NPM1*^mut^ loss diagnosis samples (*p* = 0.04, unpaired *t*-test; Fig. 6).

### *NPM1*^mut^ loss is characterized by a distinct clinical course

**Fig. 7:**
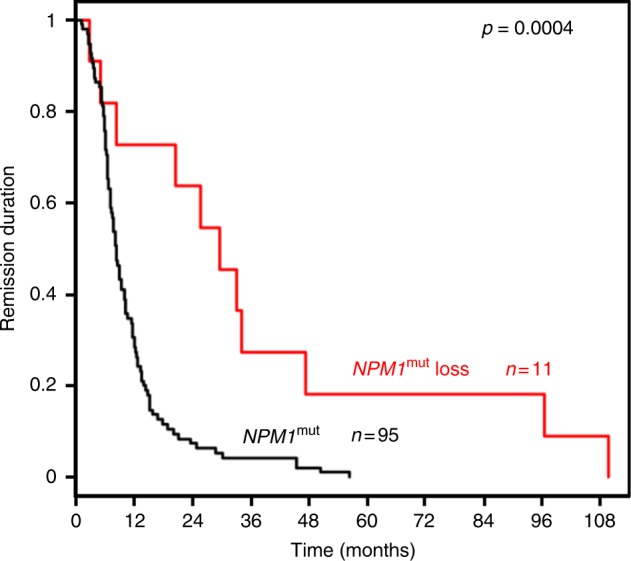
Remission duration of *NPM1*^mut^ loss and persistent pts. Pts with *NPM1*^mut^ loss (red) had a significantly longer remission duration compared to pts maintaining *NPM1*^mut^ (black) at relapse

As the mutational pattern pointed to a distinct biology in pts with *NPM1*^mut^ loss, we were interested to see whether these pts differ with regard to their clinical outcome. Indeed, *NPM1*^mut^ loss pts had a significantly longer remission duration compared to pts with persisting *NPM1*^mut^. Median time to relapse in the 11 *NPM1*^mut^ loss pts was 30 months [95% Confidence Interval (CI), 21 months—not reached] compared to 8 months in 95 pts maintaining the *NPM1*^mut^ at relapse (95% CI, 7 months—10 months; *p* = 0.0004, two-sided log-rank test; Fig. 7). In line with our previous report^7^, *NPM1*^mut^ loss pts poorly responded to salvage chemotherapy; here only 2 of 11 pts achieved a second complete remission (CR) whereas 58 of 96 (60%) *NPM1*^mut^ persistent pts with clinical data available achieved a second CR (*p* = 0.008, Fisher’s exact test).

### Possible mechanisms of relapse in *NPM1*^mut^ AML

**Fig. 8:**
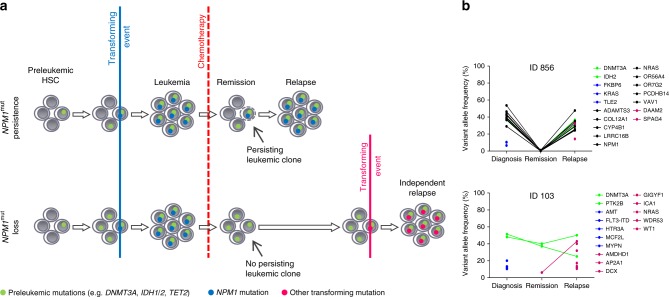
Possible mechanisms of relapse in *NPM1*^mut^ AML. **a** Based on our mutation data we postulate different mechanisms of relapse for *NPM1*^mut^ loss and *NPM1*^mut^ persistent pts. **b** Variant allele frequency plots of mutated genes from exemplary pts for each group

Based on the mutation profile, the gene expression signature, and also the clinical course of *NPM1*^mut^ loss and *NPM1*^mut^ persistent pts, two different mechanisms of relapsed disease can be discussed (Fig. 8). In *NPM1*^mut^ persistent pts an *NPM1*^mut^ leukemic clone outlasts chemotherapy, undergoes additional evolution and subsequently sprouts to cause the relapse. In contrast, in *NPM1*^mut^ loss pts the initial leukemic clone is eradicated by chemotherapy. However, dominant clonal hematopoiesis with persistence of preleukemic mutations, such as *DNMT3A*^mut^, provides the basis for the development of a second AML, caused by a transforming event different from *NPM1*^mut^ and thereby leading to a novel leukemia rather than to a relapse of the initial leukemia. This is further supported by the significantly longer remission duration in the *NPM1*^mut^ loss pts. In accordance, evaluation of VAF on average depicts persistence of preleukemic mutations in *NPM1*^mut^ loss pts, with an unchanged VAF at remission as shown in Fig. 8 (see also Supplementary Fig. 4). In contrast, in *NPM1*^mut^ persistent pts we frequently observe clearance of the leukemic clone at remission below the detection limit of our WES approach and recurrence of the mutations become apparent at relapse (Fig. 8 and Supplementary Fig. 4).

## Discussion

With regard to clonal evolution of *NPM1*^mut^ cases at the time of relapse, this extended study of 129 *NPM1*^mut^ pts confirmed our previous findings performed in a smaller cohort of *NPM1*^mut^ pts^7^ but also provides novel insights into relapse pathomechanisms of *NPM1*^mut^ AML. In general, *NPM1*^mut^ persistence was the predominant relapse mechanism of *NPM1*^mut^ AML, since we detected persisting *NPM1*^mut^ clones at relapse in 91% of pts. Moreover, detectable *NPM1*^mut^ mutation after morphological remission was accompanied by a morphological relapse, illustrating the necessity of monitoring minimal residual disease (MRD) in *NPM1*^mut^ pts. By SNP analysis we could confirm critical regions associated with disease relapse such as deletion of 12p13 leading to haploinsufficiency of *ETV6*, a tumor suppressor gene also frequently lost in AML with complex karyotypic changes^21^. Similarly, loss of 17q11.2 leads to *NF1* haploinsufficiency, which was previously shown to contribute synergistically to disease progression^22^. Relapse specific gain of 11q23.3 affects the oncogene KMT2A (MLL) whereas the mechanism of trisomy 8 still remains elusive. The frequently acquired UPD of chromosome 13 results in homozygous *FLT3*-ITD mutations. As shown in our previous study only few genomic aberrations were present in *NPM1*^mut^ diagnosis samples (0.3/patient), whereas at the time of relapse we observed a substantial increase (1/patient) in the number of genomic alterations.

Comparative mutation profiling confirmed the highest stability of *DNMT3A*^mut^ (95%), due to the early acquisition and preleukemic occurrence^11^. Similarly, other mutations associated with clonal hematopoiesis, such as *IDH1* and *IDH2* mutations, also showed a high stability (86 and 88%, respectively). These genes are involved in epigenetic regulation and mutations result in a preleukemic state of HSCs^5,10^. Preleukemic mutations confer an advantage in self-renewal and proliferation of HSCs, thereby leading to clonal expansion^23,24^. Moreover, it has been shown that *DNMT3A*^mut^ HSCs can survive induction therapy and persist at remission, thus supposedly increasing the risk for a second AML^11,25^. *NPM1*^mut^ was also a relatively stable marker, yet 9% of pts were *NPM1*^*wt*^ at relapse. In contrast *NRAS* and *FLT3* mutations were less stable, because these driver mutations are rather late events, just like other mutations in genes involved in active signaling^5,6^.

Interestingly, WES detected *PTK2B* and *PAX5* mutations persisting at the time of remission in two *NPM1*^mut^ loss pts. These mutations have not been previously described as preleukemic or germline mutations. PTK2B is a tyrosine kinase involved in the PI3K-AKT signaling pathway. PAX5 is a transcription factor, and encodes the B cell lineage specific activator protein. In another *NPM1*^mut^ loss case a mutation in BCOR, a transcriptional corepressor that interacts with histone deacetylases, hence another epigenetic modifier, was identified as preleukemic mutation. *BCORL1*, a paralog of *BCOR*, was found recurrently mutated in individuals without hematological malignancy^24^, and *BCOR* mutations have recently been linked to clonal hematopoiesis in aplastic anemia^26^ as well as secondary AML and tAML^27^. An additional potential preleukemic mutation, *NPAT*^mut^, which persisted in remission, was found in an *NPM1*^mut^ persistent patient. *NPAT* is found mutated in several cancer types including lung cancer^28^ and malignant melanoma^29^, and germline *NPAT*^mut^ was recently identified as a candidate risk factor for Hodgkin lymphoma^30^, thereby suggesting also a possible implication in AML pathogenesis.

Thus, except for preleukemic mutations the mutational profiles of *NPM1*^mut^ loss pts showed almost no overlap between diagnosis and relapse samples. This observation points to a significant difference in leukemia biology, which is also reflected by the fact that diagnosis and relapse associated gene mutations targeted different pathways. Notably, the diagnosis mutational pattern of *NPM1*^mut^ loss pts was similar to cases with *NPM1*^mut^ persistence, which further confirms *NPM1*^mut^ as disease-defining event^31^. Furthermore, it shows that *NPM1*^mut^ associated pathomechanisms lead to distinct cooperating events. In line, diagnosis and relapse mutational patterns of *NPM1*^mut^ persistent pts were also closely related and formed a cluster together with *NPM1*^mut^ loss diagnosis samples, which was separate to the ‘*NPM1*^wt^’ samples when clustering the *NPM1* associated molecular signature.

This suggests that at diagnosis *NPM1*^mut^ was the disease transforming event in both groups. In persistent pts, chemotherapy might lead to the repression and possibly elimination of the major leukemic clone, which at the time of remission cannot be detected anymore at the sensitivity level of our sequencing analysis. However, in these cases there is persistence of leukemic subclones that undergo clonal evolution and cause leukemia relapse. In *NPM1*^mut^ loss cases, chemotherapy might lead to the extinction of the leukemic clones, but persistent clonal hematopoiesis, which is dominating over healthy hematopoiesis in these cases, increases the risk for a second “de novo” AML. This hypothesis is underlined by the different mutation profiles as well as the longer duration until disease recurrence in these cases (median, 30 months vs. 8 months) since acquisition of a new driver mutation in this pre-leukemic clonal hematopoiesis setting is likely to take longer than a relapse originating from a persisting leukemic subclone. This clinical finding was recently confirmed by Höllein and colleagues^32^.

Due to limitations of WES in detecting rare variants, we conducted deep targeted sequencing of selected diagnosis samples to search for mutations in minor subclones which are present with a VAF down to 0.5%. We did not discover a relapse specific mutation at this VAF in any of the *NPM1*^mut^ loss samples, which suggests that all leukemic clones are eradicated. Moreover, the analysis of the types of point mutations revealed a higher average percentage of transversions at the time of relapse in *NPM1*^mut^ persistent compared to loss samples (45% versus 27%). This higher number of transversions in persistent pts is comparable with data reported by Ding and colleagues^17^ who also found an increase of transversions in relapse samples.

A potential explanation, why in some *NPM1*^mut^ pts the disease might be initially cured and the relapse then occurs as an independent event based on persisting clonal hematopoiesis can be derived from our gene set analyses. Interestingly, diagnostic *NPM1*^mut^ loss samples showed less stem cell-like phenotypic features due to absent enrichment of mutations in the WNT signaling pathway, a hallmark for leukemic stem cells, which acts as a regulator of self-renewal^20^. In contrast, *NPM1*^mut^ leukemias harboring more stem cell features might be more resistant to chemotherapy and thus they will eventually relapse, which in turn is characterized by the persistence of the *NPM1*^mut^. Indeed, increased WNT signaling, reflected by higher CTNNB1 levels, in *NPM1*^mut^ persistent diagnosis samples compared to *NPM1*^mut^ loss diagnosis samples was confirmed by flow cytometry analysis.

In *NPM1*^mut^ loss pts, over time there might be a selection and/or clonal evolution of a more aggressive *DNMT3A*^mut^ HSC, which results in a relapse AML also characterized by stem cell- like features. As in the general population with presence of clonal hematopoiesis this transformation process is slow and likely requires the acquisition of additional hits. Loss of *NPM1*^mut^ is a rare event and thus monitoring *NPM1*^mut^ minimal residual disease is nevertheless a valid marker for relapse prediction^33,34^. In addition, our observation further highlights the need to develop drugs which target these epigenetic regulator mutations to eradicate clonal hematopoiesis as precancerous/preleukemic lesion^35^, and even more importantly it shows the necessity to reveal the mechanism and role of preleukemic mutations.

In summary, we could show that relapsed AML of *NPM1*^mut^ loss pts possesses almost no relationship to the primary tumor so that a more suitable nomenclature would be second “de novo” AML. Further investigations are needed to evaluate whether this phenomenon can also be seen in relapses which have lost other distinct driver lesions, such as *CEBPA*^mut^, t(8;21) or inv(16). Second “de novo” AML, due to persistence of preleukemic mutations, has strong implications for the clinic since MRD monitoring has limited value in these pts. Moreover, in cases with dominant clonal hematopoiesis at the time of remission, novel treatment approaches are needed to eradicate preleukemic clones. Finally, the development of more sensitive MRD tools to detect persisting leukemic subclones will contribute to ultimately achieve cure and prevent relapse in AML pts.

## Methods

### Patients

In total, 129 *NPM1*^mut^ AML pts (25–78 years) with availability of samples at least at the time of diagnosis and at relapse were analyzed. 84% of the pts (109/129) were treated in one of four prospective AMLSG treatment trials [HD98A (*n* = 9; NCT00146120), AMLSG 07–04 (*n* = 34; NCT00151242), AMLSG 09-09 (*n* = 64; NCT00893399) and AMLSG 16-10 (*n* = 2; NCT01477606)]; the remaining 20/129 pts (16%) participated in the AMLSG BiO Registry study (NTC 01252485). All pts received intensive standard induction chemotherapy with cytarabine and an anthracycline (7 + 3 regimen) followed by high-dose cytarabine consolidation cycles with few exceptions in *NPM1*^mut^ persistence pts. Patient characteristics are summarized in Table 2. Informed consent for both treatment and biobanking of leukemia samples according to the Declaration of Helsinki was given by all pts. Approval was obtained from the ethical review board of the University of Ulm (ethical vote number 148/10).

**Table 2:** Clinical characteristics of 129 *NPM1*^mut^ patients at diagnosis

Variable	No.		%
Age, years			
Median		54	
Range		25–78	
Sex			
Female	64		50
Male	65		50
AML history			
De novo	108		84
tAML	2		2
Not available	19		15
Cytogenetics			
CN	102		79
Deletion 9q	5		4
Others	3		2
Not available	19		15
NPM1 mutation type			
A	100		78
B	12		9
D	9		7
Others	8		6
WBC count, ×10^*9*^*/L*			
Median		20	
Range		1–279	
BM Blasts, %			
Median		80	
Range		20–100	
Induction cycles, *n*			
1	16		12
2	113		88
Remission status after first induction cycle (*n* = 129)			
CR/CRi	97		75
PR	31		24
RD	1		1
Remission status after second induction cycle (*n* = 113)			
CR	112		87
Relapse	1		1
Consolidation therapy (*n* = 127)			
High-dose Cytarabine	114		88
Allogeneic SCT	9		7
Autologous SCT	4		3
Overall survival, days			
Median		535	

*CN* cytogenetically normal, *WBC* white blood cell, *BM* bone marrow, *CR* complete remission, *CRi* complete remission with incomplete hematologic recovery, *PR* partial remission, *RD* refractory disease, *SCT* stem cell transplantation

### Mutation analysis

*NPM1*^mut^ was diagnosed by locked nucleic acid (LNA)-based PCR^36^, *DNMT3A* mutation screening was performed by DNA-based amplification of the corresponding exons^37^. Subsequently, amplified DNA was used for mutational analysis by conventional Sanger sequencing to define the exact mutation types. *FLT3*-ITD, *FLT3*-TKD and *ASXL1* amplicons were screened for mutations by a GeneScan-based fragment analysis^38,39^. The amplified fragments of *IDH1*, *IDH2*, *TP53*, and *NRAS* were analyzed by denaturing high-performance liquid chromatography (DHPLC) on a WAVE 3500HT DNA Fragment Analysis System (Transgenomic)^40–42^. Samples classified as mutated were further characterized by direct sequencing. *MLL*-PTD was detected by RNA-based PCR^43^.

### Single nucleotide polymorphism microarray analysis

A total of 500 ng of purified high-quality genomic DNA (using the QIAamp DNA Mini Kit, Qiagen) from every sample was digested, ligated to adaptors, amplified by PCR, purified, fragmented, labeled and hybridized to the Genome-Wide Human SNP Array 6.0 according to manufacturer’s instructions (Affymetrix). CEL files were generated using the Command Console Software 4.1.2 (Affymetrix). The Genotyping Console Software 2.0 (Affymetrix) was used for normalization and analysis of CEL files.

### Whole-exome sequencing analysis

WES was performed on diagnosis, remission and relapse samples from 10 pts with *NPM1*^mut^ loss and 10 pts with *NPM1*^mut^ persistence at the time of relapse. DNA libraries were generated from either 500 ng DNA using the TruSeq DNA Sample Prep v2 kit (Illumina) with the TruSeq Exome Enrichment kit (Illumina) or from 50 ng DNA using the Nextera Rapid Capture Expanded Exome kit (Illumina) and the Nextera Rapid Capture Exome kit (Illumina) according to the manufacturer’s instructions. Pooled DNA libraries were sequenced on an Illumina HiSeq2000 with the 200-cycle TruSeq SBS v3 kit (Illumina). Following demultiplexing the paired-end sequences were aligned to the human reference genome hg19 with BWA-MEM^44^. BAM files were sorted and indexed using SortSam and BuildBamIndex (both Picard 1.138, http://broadinstitute.github.io/picard). To minimize the number of artefacts in downstream variant calling, PCR duplicates were removed (MarkDuplicates, Picard 1.138) and initial alignment was refined by local realignment (Indel Realigner, GATK 3.4-46)^45^. Variants were called using VarScan2 somatic^46^ by comparing tumor samples to the matching remission sample and annotated using ANNOVAR^47^. A custom script was used to rescue “germline” variants in genes known to be recurrently mutated in cancer (Supplementary Table 5). Intronic variants, synonymous variants, variants with less than 2 tumor variant supporting reads in forward and reverse direction, common SNPs >0.01% allele frequency [data base of single nucleotide polymorphisms (dbSNP Build ID138) and 1000 Genomes Project]^48,49^ without COSMIC^50^ entry (http://cancer.sanger.ac.uk), variants located in segmental duplication areas, variants located in polymer-repeat regions, and variants with a VAF <10% were filtered out. Rescued variants also present in the remission sample were assessed individually for authentic contribution to disease. Gene set analysis was performed to compute overlap between the mutated genes of each group (*NPM1*^mut^ loss diagnosis, *NPM1*^mut^ loss relapse, *NPM1*^mut^ persistent diagnosis and *NPM1*^mut^ persistent relapse) with gene sets from the Molecular Signatures Database (MSigDB, http://software.broadinstitute.org/gsea/msigdb/annotate.jsp)^51^. We selected chemical and genetic perturbations, canonical pathways, BioCarta gene sets, KEGG gene sets, Reactome gene sets, microRNA targets, transcription factor targets, and oncogenic signatures gene sets with the FDR *q*-value threshold set to 0.05. For calculation of SNV frequencies from the different mutation classes, mutations below 20% VAF were filtered out to reduce the number of false positives. Statistical significance was assessed using a nonparametric Mann-Whitney test.

### Deep amplicon sequencing

A custom amplicon panel was used to sequence selected relapse specific mutations in three *NPM1*^mut^ loss and three *NPM1*^mut^ persistent diagnosis samples with deep coverage. We used an AML custom design amplicon panel which includes 31 genes most relevant in the oncogenesis of AML. We selected six diagnosis samples to cover as many relapse specific mutations as possible with our panel. Target enrichment was performed from 50 ng DNA using the HaloPlex HS Target Enrichment System (Agilent) according to the manufacturer’s instructions. Pooled DNA libraries were sequenced on an Illumina MiSeq with the MiSeq Reagent Kit v2 (300-cycles, Illumina). One bp was trimmed from all reads, which were then aligned to the human reference genome GRCh37 using BWA-MEM^44^. Molecular barcodes were deduplicated using BamDeduplicateByBarcode from ngs-bits^52^ and alignments were sorted and indexed using Picard (Picard 1.138, http://broadinstitute.github.io/picard) and locally realigned with GATK (Indel Realigner, GATK 3.4-46)^46^. Resulting alignments were used for coverage calculation with BEDTools^53^ as well as for pileup generation and variant calling with SAMtools^54^ and VarScan2^45^ respectively. BAM files were inspected with the integrative Genomics Viewer (Broad Institute, Cambridge, MA) for presence of relapse specific mutations. HaloPlexHS is an improved next-generation targeted enrichment method allowing tracking and correction of PCR duplicates by tagging every single original DNA molecule with a molecular barcode. Using the HaloPlexHS protocol it is possible to detect rare variants down to 0.5% VAF^55^.

### RNA-Seq analysis

RNA was extracted from paired diagnosis/relapse samples of five pts with *NPM1*^mut^ loss and five pts with *NPM1*^mut^ persistence using the AllPrep DNA/RNA Mini Kit (QIAGEN), and RNA quality was assessed using a BioAnalyzer 2100 (Agilent). Libraries were prepared from 1 µg of total RNA using the TruSeq Stranded Total RNA Kit with Ribo-Zero Human/Mouse/Rat (Illumina) according to the manufacturer’s instructions. The pooled RNA libraries were sequenced on an Illumina HiSeq2000 to obtain 100 bp paired-end reads. RNA-Seq reads were aligned to the human reference genome hg 19 and quantified using STAR v.2.4.2a^56^ in the 2-pass mapping mode. Furthermore, the DESeq2^57^ package from R was used to obtain normalized expression values. BRB-ArrayTools Version 4.5.0 Beta_2^58^ (BRB, National Cancer Institute, Bethesda, MD, USA) and GSEA (http://broadinstitute.org/gsea/index.jsp)^51^ were used for class comparison analyses. Hierarchical clustering and heatmap visualization of differentially expressed genes was performed using Cluster 3.0^59^ and Java Treeview^60^.

### Flow cytometry

Intracellular flow cytometry was performed using the Fix & Perm Cell Permeabilization Kit (Invitrogen)^61^. In brief, primary patient cells were resuspended in 100 µl Fixation Medium and incubated for 15 min at room temperature. Cells were then washed in 3 ml PBS + 0.1% NaN_3_ + 5% FBS followed by centrifugation. Staining with the respective antibodies and isotope controls was performed in 100 µl of the permeabilization medium according to the manufacturer’s instructions for 1 h at room temperature followed by washing and centrifugation as indicated above. We used a primary antibody against total CTNNB1 (anti-h-beta-Catenin, APC-conjugated, #IC13292A, R&D Systems) or active CTNNB1 (anti-active-b-Catenin, clone 8E7, #05-665-25UG, Merck Millipore) followed by a secondary Alexa488 labeled antibody (donkey anti-mouse, Invitrogen # R37114). Cells were analyzed using a CantoII^TM^ (Becton-Dickinson) cytometer. Data analysis was performed using FlowJo^TM^ software (Treestar, Ashland, OR).

### Statistical analyses

Statistical analyses for clinical outcome were performed as follows: The definition of CR, remission duration and overall survival (OS) were based on recommended criteria from the 2017 ELN recommendations^4^. Remission duration endpoints measured from the date of documented CR were relapse (failure), death in CR (censored) and alive in CR at last follow-up (censored). The Kaplan–Meier method was used to estimate distributions of remission duration and differences between two groups were analyzed using the two-sided log-rank test. Response to salvage therapy was analyzed using Fisher’s exact test and expression data with normal distribution by unpaired t-test. An effect was considered significant if *P* ≤ 0.05. All statistical analyses were performed with the statistical software environment R version 3.0.2, using the R packages Hmisc version 3.13-0 and survival version 2.37-7^62^.

### Reporting summary

Further information on research design is available in the Nature Research Reporting Summary linked to this article.

## Supplementary information

Supplementary Information

Description of Additional Supplementary Files

Supplementary Data 1

Supplementary Data 2

Supplementary Data 3

Supplementary Data 4

Supplementary Data 5

Reporting Summary

## Data Availability

SNP microarray data and RNA-Seq data that support the findings of this study have been deposited in the NCBI Gene Expression Omnibus^63^ (GEO) and are accessible through GEO Series accession numbers GSE46951 and GSE128604. WES raw data that support the findings of this study are available from the corresponding author upon reasonable request. Evaluated true variants from WES and deep amplicon sequencing are provided within the paper and its supplementary information files.
