# Supplementary material for: Clonal evolution patterns in acute myeloid leukemia with NPM1 mutation
Source: Nat Commun. 2019 May 2;10:2031. doi: 10.1038/s41467-019-09745-2 (PMC6497712; doi:10.1038/s41467-019-09745-2)
Supplement: Supplementary file 1 — Supplementary Information [file 41467_2019_9745_MOESM1_ESM.pdf]

Supplementary Information

Clonal evolution patterns in acute myeloid leukemia with *NPM1* mutation

Cocciardi et al.

Supplementary Figures

Supplementary Figure 1

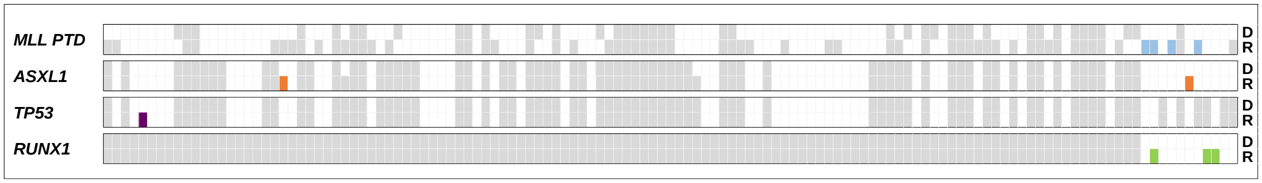

Incidence of *MLL*-PTD, *ASXL1*, *TP53* and *RUNX1* mutations in 129 paired (diagnosis/relapse) *NPM1*^{mut} pts. Colored bars indicate the presence of a mutation, white bars represent wild-type, data not available is indicated by a grey bar; D, diagnosis; R, relapse.

Supplementary Figure 2

a

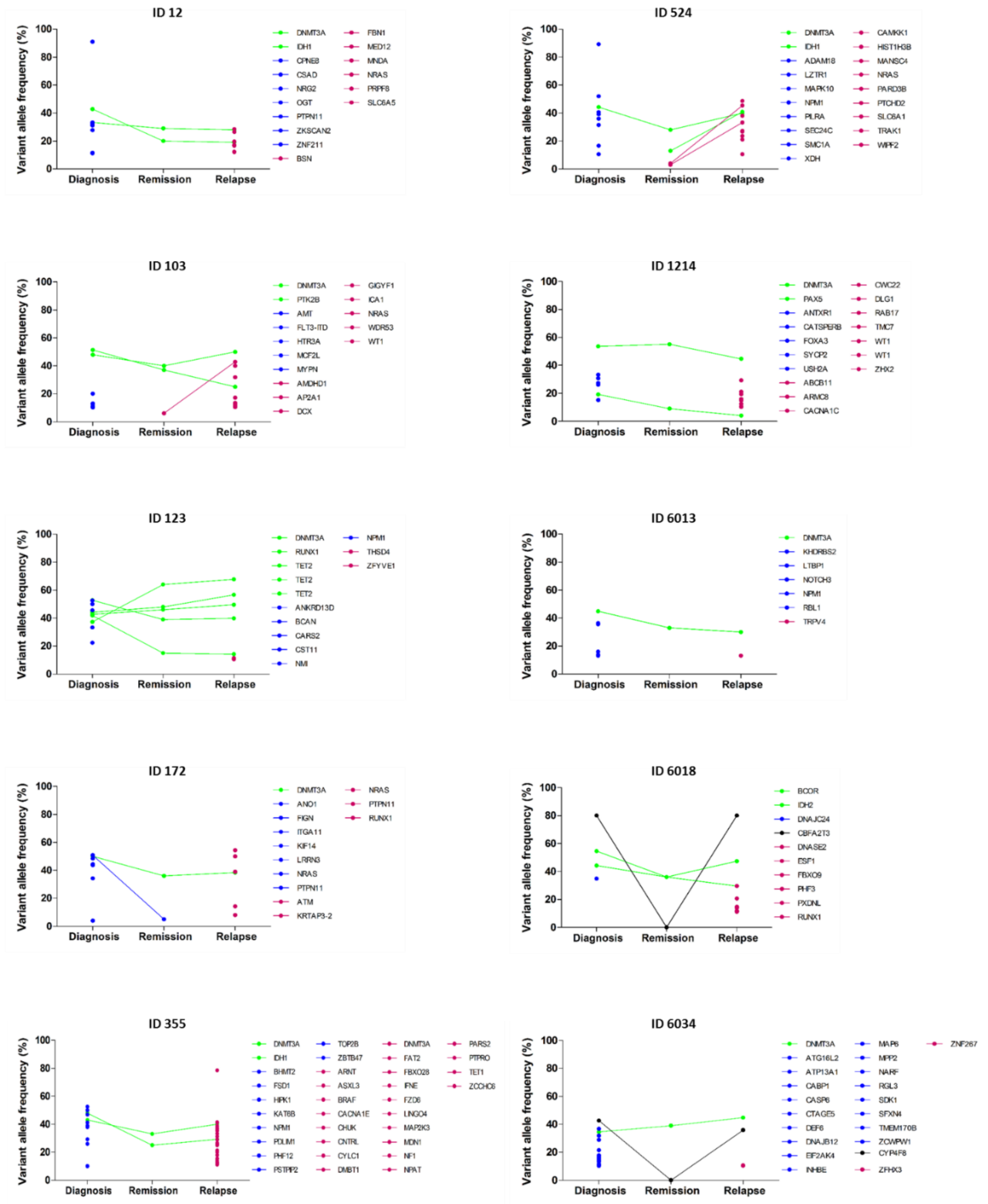

b

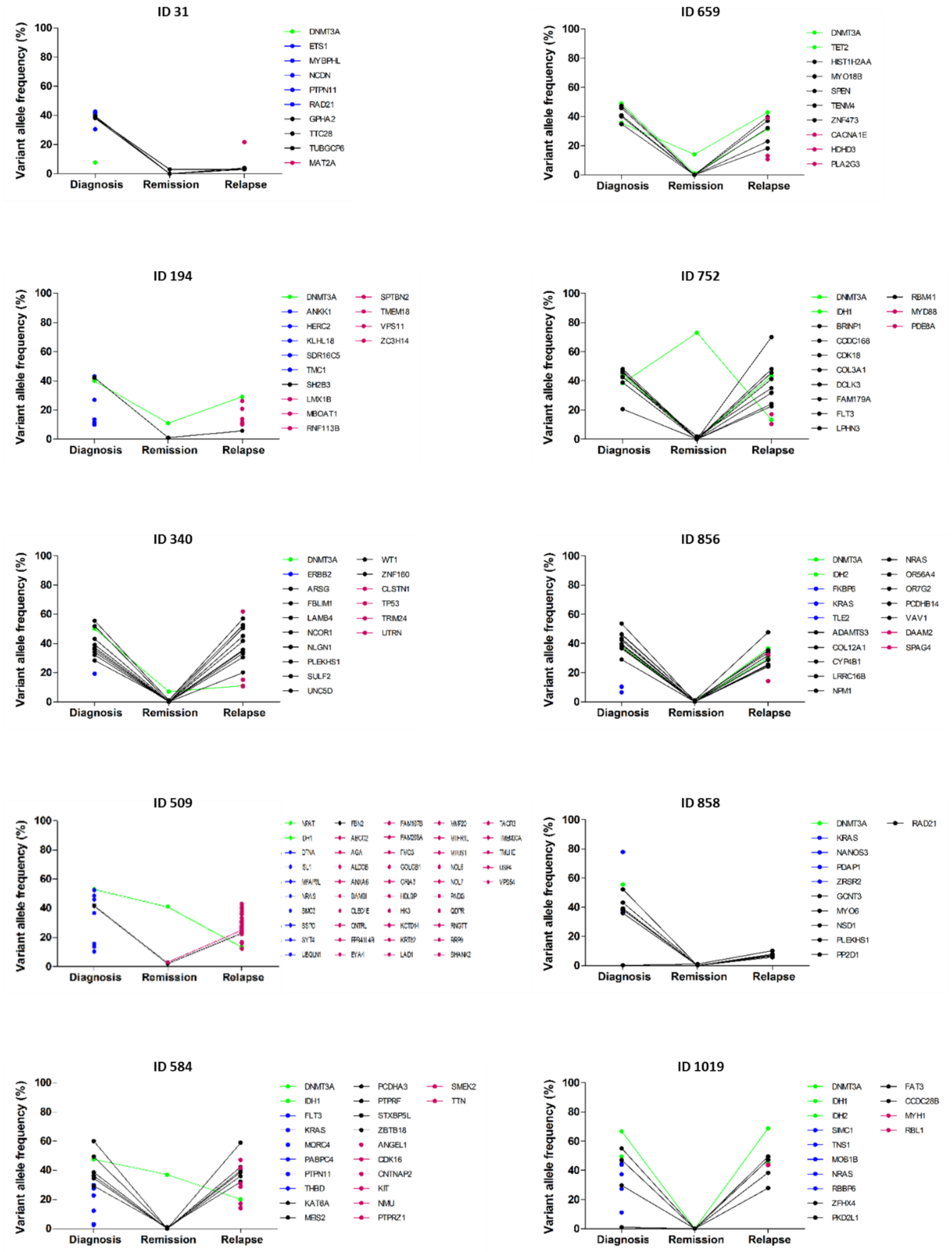

VAF plots of mutations detected with WES. The VAF progression is depicted from diagnosis to relapse in (**a**) *NPM1*^{mut} loss pts and (**b**) *NPM1*^{mut} persistent pts. Mutations in green represent preleukemic mutations, mutations in blue are specific to the diagnosis, mutations in red are specific to the relapse and mutations in black are shared between diagnosis and relapsed sample.

Supplementary Figure 3

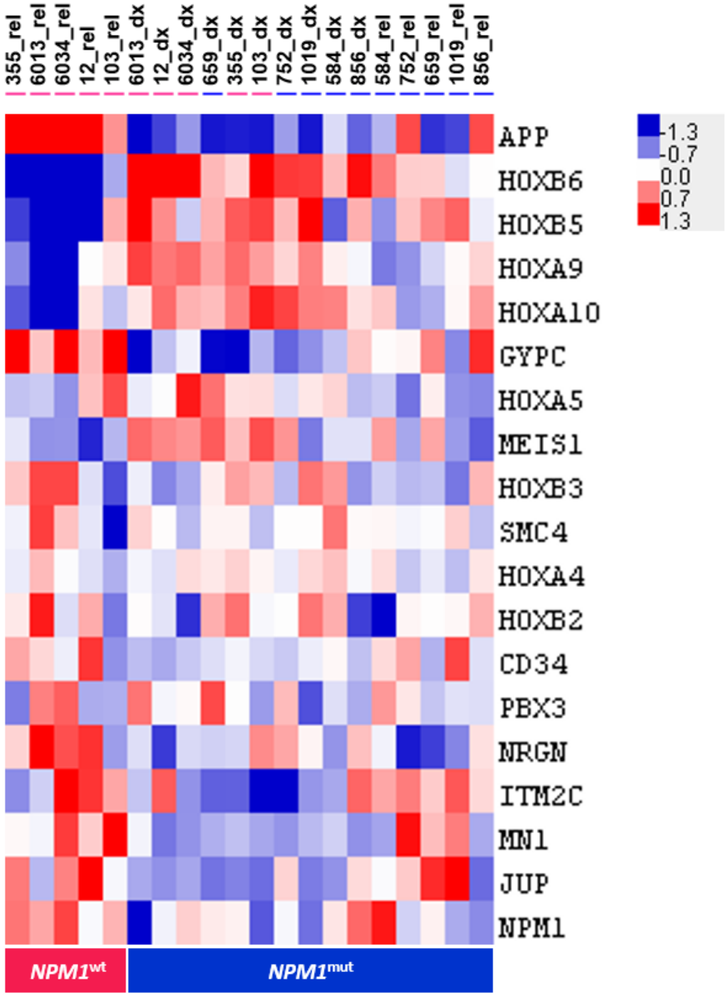

Heatmap of genes predictive of *NPM1*^{mut} status found by Verhaak and colleagues. Clustering of 18 *NPM1* molecular signature genes and *NPM1* of paired diagnosis and relapse samples from 5 *NPM1*^{mut} loss and 5 *NPM1*^{mut} persistent pts (n=20). The signature is lost in NPM1^{mut} loss relapse samples (n=5), which are *NPM1*^{wt} and group together. Red lines below the ID indicate *NPM1*^{mut} loss samples, blue lines indicate *NPM1*^{mut} persistent samples; rel, relapse; dx, diagnosis.

Supplementary Figure 4

a

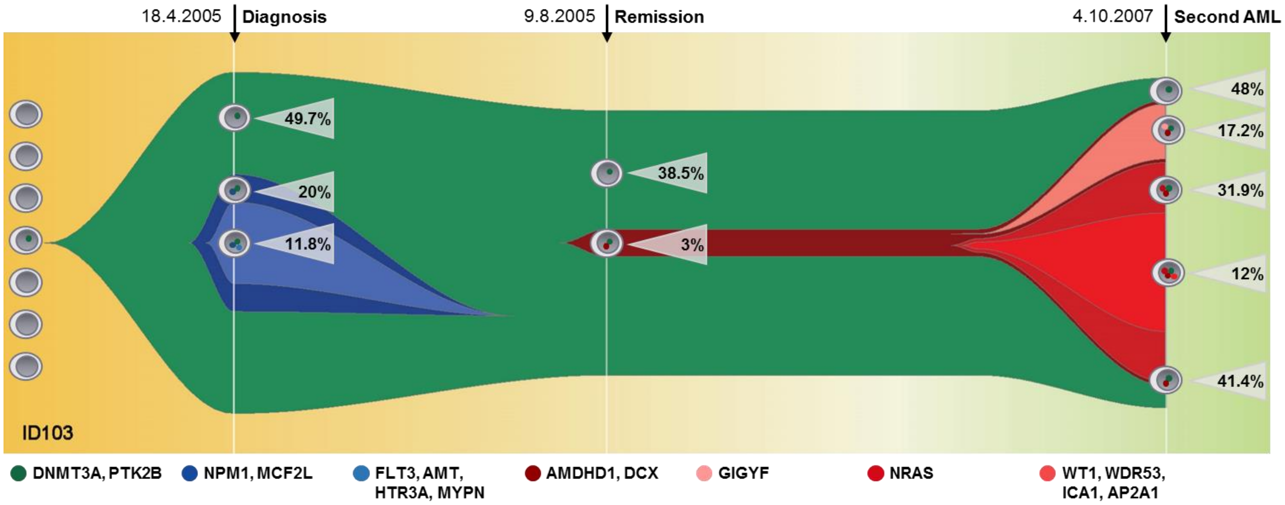

b

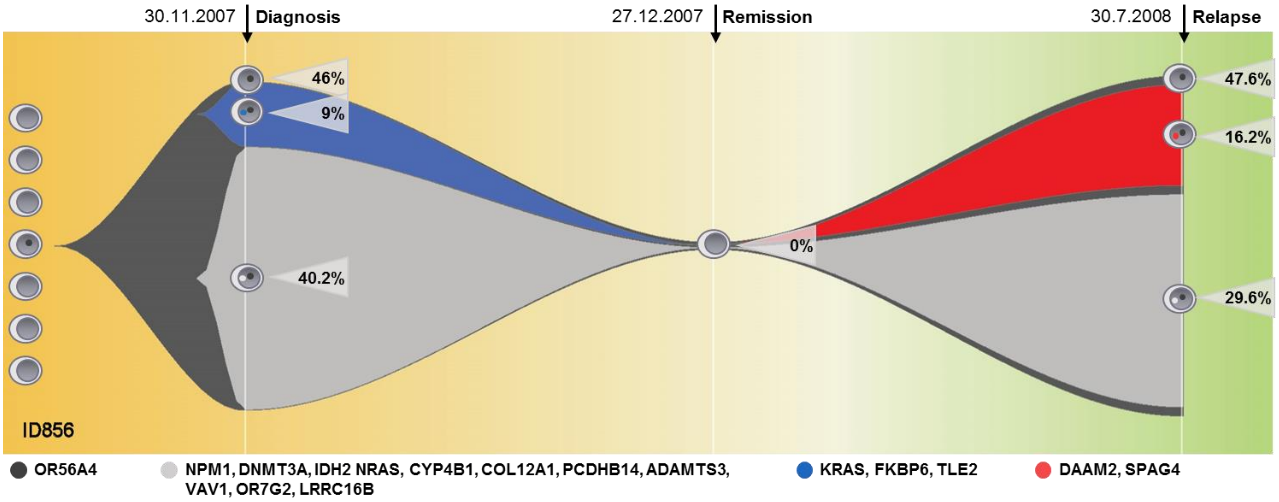

Fishplots of a representative *NPM1*^{mut} loss and *NPM1*^{mut} persistent patient. (**a**) Depicted is the clonal evolution of *NPM1*^{mut} loss patient ID103 with the characteristic loss of all diagnosis specific mutations (clones in blue shades), but persistence of the preleukemic clone (green) and acquisition of new mutations at relapse (clones in red shades) after a period of 20 months in remission. (**b**) Depicted is the clonal evolution of *NPM1*^{mut} persistent patient ID856 with the leukemic clones (in grey shades), comprising the *NPM1*^{mut}, reappearing at relapse. One diagnosis subclone (blue) is lost, while a new one appears at relapse (red). Remission duration for this patient was 7 months.

Supplementary Tables

Supplementary Table S1

Incidence of recurrent mutations.

Gene	Mutated at diagnosis/ Patients screened at diagnosis	Loss at relapse	Gain at relapse
NPM1	129 / 129	11	0
DNMT3A	83 / 129	4	1
FLT3-ITD	40 /129	10	23
IDH1	29 / 128	4	4
IDH2	24 / 129	3	1
NRAS	23 / 128	15	7
FLT3-TKD	22 /129	16	1
ASXL1	0 / 61	0	2
TP53	0 / 54	0	1
MLL-PTD	0 / 87	0	4
RUNX1	0 / 11	0	3

Supplementary Table 2

Mutation shift of 10 recurrently mutated genes in 129 AML samples.

No. of mutations shifted	in *NPM1*^{mut} loss pts (n=11)	in *NPM1*^{mut} persistent pts (n=118)
0	0	53
1	3	51
2	5	11
3	1	3
4	1	0
5	1	0

Supplementary Table 3

CNA at diagnosis and relapse

Aberration	Candidate genes in CDR	Diagnosis	Lost	Acquired	Relapse
del(9)(q21)	HNRNPK, NTRK2	6	3		3
UPD(13q)	FLT3	4	1	11	14
UPD(6p)	?	3	2		1
UPD(1p)	?	1	1		0
UPD(2p)	?	1			1
Nonrecurrent CNAs at diagnosis		8	4		4
del(12)(p13)	ETV6	0		4	4
del(4)(q22.1)	CCSER1	0		3	3
gain(11)(q23.3)	KMT2A	0		3	3
del(11)(p13)	WT1	0		3	3
del(17)(q11.2)	NF1			3	3
Trisomy 8				3	3
del(3)(p14.2)	FHIT	0		2	2
del(7)(p15.1p14.3)	?	0		2	2
del(10)(p14p12.31)	?	0		2	2
del(13)(q13.3)	?			2	2
del(14)(q21.1)	?			2	2
del(17)(p13p11.2)	TP53			2	2
Trisomy 5	?			1	1
Monosomy 7	?			1	1
-X	?			1	1
UPD(22q)				1	1
Nonrecurrent CNAs at relapse				21	21
**Total**		**23**	**11**	**67**	**79**

Abbreviations: CNA, Copy number aberration; CDR, Coding DNA region; UPD, Uniparental disomy; del, deletion

Supplementary Table 4

Coverage of WES of 20 paired diagnosis, remission and relapse samples from *NPM1*^{mut} loss and persistent AML patients.

ID	Sample Type	Mean coverage	1x Coverage	10x Coverage	50x Coverage
NPM1^{mut} loss					
12	Diagnosis	84	95%	76%	50%
	Remission	86	94%	77%	54%
	Relapse	81	93%	74%	49%
103	Diagnosis	69	92%	70%	40%
	Remission	76	93%	73%	42%
	Relapse	102	96%	82%	53%
123	Diagnosis	74	96%	91%	66%
	Remission	89	96%	92%	74%
	Relapse	47	94%	81%	34%
172	Diagnosis	51	92%	73%	41%
	Remission	65	95%	90%	59%
	Relapse	32	93%	69%	19%
355	Diagnosis	57	96%	90%	54%
	Remission	30	92%	64%	20%
	Relapse	51	96%	89%	45%
524	Diagnosis	51	96%	89%	46%
	Remission	63	96%	90%	60%
	Relapse	48	93%	75%	37%
1214	Diagnosis	93	94%	74%	51%
	Remission	100	94%	77%	56%
	Relapse	92	95%	76%	53%
6013	Diagnosis	94	99%	90%	63%
	Remission	57	98%	84%	44%
	Relapse	45	91%	66%	32%
6018	Diagnosis	71	94%	74%	42%
	Remission	86	95%	79%	48%
	Relapse	77	94%	75%	43%
6034	Diagnosis	86	94%	76%	47%
	Remission	50	89%	63%	31%
	Relapse	75	91%	69%	41%

ID	Sample Type	Mean coverage	1x Coverage	10x Coverage	50x Coverage
NPM1^{mut} persistent					
31	Diagnosis	122	97%	85%	62%
	Remission	122	96%	84%	63%
	Relapse	147	97%	88%	71%
194	Diagnosis	50	93%	73%	38%
	Remission	121	97%	85%	63%
	Relapse	154	98%	89%	71%
340	Diagnosis	76	95%	78%	50%
	Remission	149	97%	86%	67%
	Relapse	58	94%	74%	42%
509	Diagnosis	168	97%	87%	69%
	Remission	80	93%	76%	50%
	Relapse	145	100%	97%	82%
584	Diagnosis	144	97%	84%	65%
	Remission	132	97%	86%	65%
	Relapse	130	97%	85%	63%
856	Diagnosis	137	100%	97%	80%
	Remission	130	100%	97%	79%
	Relapse	136	100%	97%	80%
858	Diagnosis	133	97%	85%	63%
	Remission	127	98%	89%	67%
	Relapse	93	97%	83%	57%
1019	Diagnosis	125	96%	83%	61%
	Remission	119	95%	80%	59%
	Relapse	113	96%	83%	60%
659	Diagnosis	69	93%	76%	47%
	Remission	62	94%	76%	45%
	Relapse	79	95%	83%	57%
752	Diagnosis	68	93%	75%	46%
	Remission	90	96%	80%	54%
	Relapse	64	96%	80%	46%

Supplementary Table 5

Rescue list for recurrently mutated genes in cancer which are graded "germline" and subsequently are filtered out.

Rescued AML genes		
ABL1	GATA2	PCBP1
ABL2	GIGYF2	PCBP2
ARID5B	GNAS	PDGFRA
ASXL1	HNRNPK	PDGFRB
ATRX	HOXA	PHF6
BCOR	HRAS	PTEN
BCORL1	IDH1	PTK2B
BRAF	IDH2	PTPN1
CALR	IKZF1	PTPN11
CBL	IKZF3	RAD21
CBLB	IL2RB	ROS1
CBLC	IL7R	RUNX1
CDKN2A	JAK1	SETBP1
CDKN2B	JAK2	SF3B1
CEBPA	JAK3	SH2B3
CRLF2	KDM6A	SMC1A
CSF1R	KIT	SMC3
CSF3R	KRAS	SRSF2
CUX1	LEF1	STAG2
DGHK	MLL	TCF3
DNMT3A	MPL	TEL
DYRK1A	MYD88	TET2
EBF1	NF1	TP53
EPOR	NOTCH1	TSLP
ETV6	NPAT	TYK2
EZH2	NPM1	U2AF1
FBXW7	NRAS	U2AF35
FLT3	NTRK3	WT1
GATA1	PAX5	ZRSR2
