# Supplementary material for: Clonal evolution patterns in acute myeloid leukemia with NPM1 mutation
Source: Nat Commun. 2019 May 2;10:2031. doi: 10.1038/s41467-019-09745-2 (PMC6497712; doi:10.1038/s41467-019-09745-2)
Supplement: Supplementary file 2 — Description of Additional Supplementary Files [file 41467_2019_9745_MOESM2_ESM.pdf]

Description of Additional Supplementary Information

File Name: Supplementary Data 1
Description: Mutations found by WES in diagnosis, remission and relapse samples from NPM1mut loss AML patients.

File Name: Supplementary Data 2
Description: Mutations found by WES in diagnosis, remission and relapse samples from NPM1mut persistent AML patients.

File Name: Supplementary Data 3
Description: Selected significantly enriched MSigDB gene sets for mutated genes in diagnosis and relapse samples from AML patients with NPM1mut loss or persistence.

File Name: Supplementary Data 4
Description: Differentially expressed genes between diagnosis and relapse samples from NPM1mut loss AML patients.

File Name: Supplementary Data 5
Description: Selected enriched pathways in NPM1mut loss samples.
