# Supplementary material for: Clonal evolution patterns in acute myeloid leukemia with NPM1 mutation
Source: Nat Commun. 2019 May 2;10:2031. doi: 10.1038/s41467-019-09745-2 (PMC6497712; doi:10.1038/s41467-019-09745-2)
Supplement: Supplementary file 8 — Reporting Summary [file 41467_2019_9745_MOESM8_ESM.pdf]

Corresponding author(s): Lars Bullinger
Last updated by author(s): Mar 18, 2019

Reporting Summary

Nature Research wishes to improve the reproducibility of the work that we publish. This form provides structure for consistency and transparency in reporting. For further information on Nature Research policies, see Authors & Referees and the Editorial Policy Checklist.

Statistics

For all statistical analyses, confirm that the following items are present in the figure legend, table legend, main text, or Methods section.

n/a	Confirmed	
☐	☒	The exact sample size (*n*) for each experimental group/condition, given as a discrete number and unit of measurement
☐	☒	A statement on whether measurements were taken from distinct samples or whether the same sample was measured repeatedly
☐	☒	The statistical test(s) used AND whether they are one- or two-sided<br>*Only common tests should be described solely by name; describe more complex techniques in the Methods section.*
☐	☒	A description of all covariates tested
☐	☒	A description of any assumptions or corrections, such as tests of normality and adjustment for multiple comparisons
☐	☒	A full description of the statistical parameters including central tendency (e.g. means) or other basic estimates (e.g. regression coefficient) AND variation (e.g. standard deviation) or associated estimates of uncertainty (e.g. confidence intervals)
☐	☒	For null hypothesis testing, the test statistic (e.g. *F*, *t*, *r*) with confidence intervals, effect sizes, degrees of freedom and *P* value noted<br>*Give P values as exact values whenever suitable.*
☒	☐	For Bayesian analysis, information on the choice of priors and Markov chain Monte Carlo settings
☐	☒	For hierarchical and complex designs, identification of the appropriate level for tests and full reporting of outcomes
☒	☐	Estimates of effect sizes (e.g. Cohen's *d*, Pearson's *r*), indicating how they were calculated

Our web collection on statistics for biologists contains articles on many of the points above.

Software and code

Policy information about availability of computer code

Data collection	SNP-Microarrays: Command console software (V4.1.2, Affymetrix). High-throughput-sequencing: BaseSpace (Illumina); MiSeq Reporter v2.6 (Illumina)
Data analysis	SNP-Microarrays: Aroma.affymetrix R package in combination with the DNAcopy R package using R version 3.2.1.; Genotyping Console software v4.2 (Affymetrix). High-throughput-sequencing: BWA-MEM; BamDeduplicateByBarcode (ngs-bits); SortSam, BuildBamIndex, MarkDuplicates (Picard 1.138); Indel Realigner (GATK 3.4-46); BEDTools; SAMtools; VarScan2; ANNOVAR; STAR v.2.4.2a; DESeq2 package from R; BRB-ArrayTools Version 4.5.0 Beta_2 (National Cancer Institute); GSEA (Broad Institute); Cluster 3.0; Java Treeview. Flow Cytometry: FlowJoTM software (Treestar)

For manuscripts utilizing custom algorithms or software that are central to the research but not yet described in published literature, software must be made available to editors/reviewers. We strongly encourage code deposition in a community repository (e.g. GitHub). See the Nature Research guidelines for submitting code & software for further information.

Data

Policy information about availability of data

All manuscripts must include a data availability statement. This statement should provide the following information, where applicable:

- Accession codes, unique identifiers, or web links for publicly available datasets
- A list of figures that have associated raw data
- A description of any restrictions on data availability

SNP microarray data and RNA-Seq data that support the findings of this study are available from Gene Expression Omnibus (GEO) (identifiers are provided in the data availability statement). WES raw data that support the findings of this study are available from the corresponding author upon reasonable request. Evaluated true variants from WES and deep amplicon sequencing are provided within the paper and its supplementary information files.

Field-specific reporting

Please select the one below that is the best fit for your research. If you are not sure, read the appropriate sections before making your selection.

☒ Life sciences ☐ Behavioural & social sciences ☐ Ecological, evolutionary & environmental sciences

For a reference copy of the document with all sections, see nature.com/documents/nr-reporting-summary-flat.pdf

Life sciences study design

All studies must disclose on these points even when the disclosure is negative.

Sample size	Sample size was determined by availability of AML patients with NPM1 mutation at diagnosis and availablity of diagnosis and relapse samples of these patients.
Data exclusions	No data was excluded from the analysis.
Replication	We have biological replicates included, for details please see the manuscript and rebuttal letters.
Randomization	Samples were allocated according to their NPM1 mutation status at relapse. We studied the differences between patients that have a persistent NPM1 mutation at relapse and patients that have lost the NPM1 mutation at relapse.
Blinding	Blinding was not relevant to our study as we were interested in the differences of the two groups (NPM1mut persistant vs NPM1mut loss).

Reporting for specific materials, systems and methods

We require information from authors about some types of materials, experimental systems and methods used in many studies. Here, indicate whether each material, system or method listed is relevant to your study. If you are not sure if a list item applies to your research, read the appropriate section before selecting a response.

Materials & experimental systems

n/a	Involved in the study	
☐	☒	Antibodies
☒	☐	Eukaryotic cell lines
☒	☐	Palaeontology
☒	☐	Animals and other organisms
☐	☒	Human research participants
☐	☒	Clinical data

Methods

n/a	Involved in the study	
☒	☐	ChIP-seq
☐	☒	Flow cytometry
☒	☐	MRI-based neuroimaging

Antibodies

Antibodies used	We used a primary antibody against total CTNNB1 (anti-h-beta-Catenin, APC-conjugated, #IC13292A, R&D Systems) or active CTNNB1 (anti-active-b-Catenin, clone 8E7, #05-665-25UG, Merck Millipore) followed by a secondary Alexa488 labeled antibody (donkey anti-mouse, Invitrogen # R37114).
Validation	Anti-h-beta-Catenin, APC-conjugated Antibody: Validated in previous publications (Gandillet et al. Heterogeneous sensitivity of human acute myeloid leukemia to β-catenin down-modulation. Leukemia 2011; Heidel, F. H. et al. Genetic and pharmacologic inhibition of β-catenin targets imatinib-resistant leukemia stem cells in CML. Cell Stem Cell 2012.)<br>anti-active-b-Catenin, clone 8E7: Validation statement on the manufacturers website; Flow Cytometry (FC):Representative lot data. An optimal 1 µg/mL concentration of this antibody was used in flow cytometry. Green indicates FITC conjugated mouse IgG1 isotype control (cat. # AP308F). Purple indicates anti-active beta catenin, clone 8E7 (cat. # 05-665) and validated in previous publications (Doglioni et al. (2003). Am J Pathol. 163: 2277-87.)<br>Donkey anti-Mouse IgG (H+L) ReadyProbes™ Secondary Antibody, Alexa Fluor 488: Validated in previous publications (Heidel, F. H. et al. Genetic and pharmacologic inhibition of β-catenin targets imatinib-resistant leukemia stem cells in CML. Cell Stem Cell 2012.)

Human research participants

Policy information about studies involving human research participants

Population characteristics	All the requested information is provided in the manuscript, please see Table 2: Clinical characteristics of 129 NPM1mut patients at diagnosis.

Recruitment	Patients were selected based on their mutational status of NPM1 at diagnosis.
Ethics oversight	Ethical review board of the University of Ulm (ethical vote number 148/10).

Note that full information on the approval of the study protocol must also be provided in the manuscript.

Clinical data

Policy information about clinical studies
All manuscripts should comply with the ICMJE guidelines for publication of clinical research and a completed CONSORT checklist must be included with all submissions.

Clinical trial registration	NCT00146120, NCT00151242, NCT00893399, NCT01477606 and 01252485. Please also see the manuscript for more information.
Study protocol	Not relevant to the analyis performed in this study.
Data collection	We performed a retrospective analysis of biobanked samples. This study was conducted between 2016 and 2019.
Outcomes	The trial is not reporting on a clinical study, but is a companion study focusing on molecular changes underlying leukemia.

Flow Cytometry

Plots

Confirm that:

☒ The axis labels state the marker and fluorochrome used (e.g. CD4-FITC).

☒ The axis scales are clearly visible. Include numbers along axes only for bottom left plot of group (a 'group' is an analysis of identical markers).

☒ All plots are contour plots with outliers or pseudocolor plots.

☒ A numerical value for number of cells or percentage (with statistics) is provided.

Methodology

Sample preparation	Intracellular flow cytometry was performed using the Fix & Perm Cell Permeabilization Kit (Invitrogen). In brief, primary patient cells were resuspended in 100 µl Fixation Medium and incubated for 15 minutes at room temperature. Cells were then washed in 3 ml PBS + 0.1% NaN3 + 5% FBS followed by centrifugation. Staining with the respective antibodies and isotope controls was performed in 100 µl of the permeabilization medium according to the manufacturer's instructions for 1 hour at room temperature followed by washing and centrifugation as indicated above.
Instrument	CantoIITM (Becton-Dickinson) cytometer
Software	FlowJoTM software (Treestar)
Cell population abundance	Analysis was performed on Ficoll enriched blast populations with a purity of at least 90%.
Gating strategy	The gating strategy was performed as previously described (Heidel, F. H. et al. Genetic and pharmacologic inhibition of β-catenin targets imatinib-resistant leukemia stem cells in CML. Cell Stem Cell 2012)

☐ Tick this box to confirm that a figure exemplifying the gating strategy is provided in the Supplementary Information.
